# Sex Education and Comprehensive Health Education in the Future of Educational Professionals

**DOI:** 10.3390/ijerph20043296

**Published:** 2023-02-13

**Authors:** Francisco Javier Jiménez-Ríos, Gracia González-Gijón, Nazaret Martínez-Heredia, Ana Amaro Agudo

**Affiliations:** Department of Pedagogy, University of Granada, 18071 Granada, Spain

**Keywords:** comprehensive sexual education, health, young people, university, Spain

## Abstract

This work shows an approach to comprehensive sexual education as an anthropological key to promoting health in the self-realisation of future education professionals. Comprehensive sexual education and health form a system. To carry out this study, we have analysed the opinion that students of the Faculty of Education Sciences of the University of Granada (Spain) have about the comprehensive sexual education received and the importance of this training in their professional practice. For this purpose, we used a quantitative and exploratory research design, using a questionnaire as an instrument for collecting information with a sample of 293 students. The results show that students have received poor sex education, along with the belief that education professionals do not receive proper and organised training in sex education. We can conclude that the majority of respondents consider sex education to be a recognised right, highlighting the importance of education professionals receiving proper training in sex education at university, where content related to respect, education for equality, and sexual health is prioritised. Sexuality constitutes the fundamental anthropological structure: comprehensive sexual education is a source of personal (corporal, psychic, spiritual) and social health, hence the importance of educating in comprehensive sexuality.

## 1. Introduction

At present, sexual education continues to be a pending subject in many countries [1,2,3,4]. Access to this knowledge base is adjusted to each educational stage, emphasising personal development with a scientific basis, but access that is gradual and appropriate to the context and stage of development is insufficient and educational institutions and bodies dedicated to this subject encounter difficulties and obstacles to its teaching [3,5,6].

Even so, the definition of sexual education has changed over the years and is now defined as a pedagogical activity, adapted to each age and culture, which uses rigorous, realistic, and unbiased scientific information at the biological, psychological, and social levels to educate about sexuality, understood as human communication, a source of health, pleasure, and affection [2,7].

Sexual education is a right, recognised by the United Nations [7,8,9,10] and prioritised in the Sustainable Development Goals under the goal of health and well-being [11], to build a society in which women and men can live in equality and without discrimination, ensuring healthy lives and promoting well-being at all ages [7]. The World Health Organization [6] defines sexual health as a state of physical, mental, and social well-being concerning sexuality, and not merely the absence of disease or infirmity. Thus, the goal of sexual education should be the enhancement of personal life and relationships, respecting and protecting sexual rights, and not only care and counselling regarding pregnancy or the prevention of sexually transmitted infections (STIs).

There have been numerous international and national implications for sexual education and health. In the document “Promoting sexual health” [9], sexual rights, which emerged in Valencia in 1997, are shown to be at the core of comprehensive sexual education and the basis for a healthy society. It mentions that sexual education is key to a healthy life. Similarly, in the Montreal Declaration [12], sexual rights education is again taken up as a key to health, to the extent that the World Association of Sexology is renamed the World Association for Sexual Health.

At the Punta Cana congress [13], the Latin American Federation of Societies of Sexology and Sexual Education culminates with a declaration that launches with new impetus the previous contributions: “Comprehensive sexual education”. Sexual and mental health education is fundamental for physical, personal, and social health. In Asunción, shortly after the promulgation of the document “Promotion of sexual health”, Flores-Colombino [14] insisted in a very direct discourse on the importance of implementing local and global mechanisms that facilitate the exercise of sexual education as a way to sexual health, a path to human health. Likewise, in Spain, in 2011, in the context of scientifically based sexual education, an energetic commitment was made, which crystallised in the document “National Strategy for Sexual and Reproductive Health” [15], which was taken up again in 2019 as an operational plan, “Sexual Health Strategy” [16].

Thus, in our country, the Spanish Constitution promotes “the right to the full development of the human personality” (art. 27.2) and the “right to the protection of health” (art. 43.1). Since the implementation of the LOGSE [17], affective-sexual education has been part of the comprehensive sexual education of pupils and is therefore socially recognised and legitimised as another right. Since then, the different educational reforms have modified aspects of its treatment and the curriculum, but without questioning the need to deal with these issues in the educational context.

The current education law, LOMLOE [18], repeatedly mentions the need to provide quality affective-sexual education that addresses issues such as desire orientation, sexual diversity and identity, gender, etc., but teachers are not being provided with the necessary training to integrate these issues in the primary classroom. However, at the curricular level, sexual education is not incorporated as a subject or as subject-specific content. There are isolated contents and topics, but it is not included in the educational curriculum or textbooks [2,4].

All human behaviour is influenced by culture and by values that are learned and developed in the socio-cultural sphere, where both the family and the school play a predominant role [19,20]. Sexual behaviour is part of this process and, therefore, must have a specific and important weight in the comprehensive sexual education of children and young people as a suitable learning tool. Sexual education helps to prepare young people for life in general, especially to build and maintain satisfactory relationships that contribute to the positive development of personality and self-esteem [1,3].

We start from a positive conceptualisation of health, defining sexuality as a fundamental human dimension for personal well-being through its different possibilities (pleasure, affection, communication, procreation) that can be experienced in relation to different degrees of commitment and with an erotophilic attitude (positive towards sexuality) and tolerant of sexual diversity [3].

Each person, as an open reality, is constituted in a process of realisation oriented towards a healthy life. A process occurs in which the relationship with other people, in the world, is fundamental, as evidenced by personalism [21] or the experiences in which one has grown up without the warmth of other people. Education, exclusive to human personal reality [22], is shown to be the privileged place for the realisation of that healthy life that constitutes the end of our process of realisation [23].

Given that human personal reality is a communicative and symbolic reality, sexuality constitutes the fundamental anthropological structure, insofar as it shows our whole world of personal relations. Thus, education and health form a system, and comprehensive sexual education and health show the apex of this system [19,24], a system in which the epistemological principality is of education and the ontological principality is of health. The only reality that is an end in itself is the personal human reality. Education is the privileged means of health, personal, and social health being the end and the means of personal and historical self-realisation. This would be the dynamic core of a theory of sexual education. The education-health system constitutes a fundamental anthropological key [25,26].

We need, therefore, training that allows students to replace conceptions based on prejudices and unfounded beliefs with rigorous knowledge. This includes assuming natural attitudes towards sexuality and understanding it as a form of human communication and a source of health, pleasure, affection, and, when desired, reproduction [1,25]. With these premises of sexual education as a source of comprehensive health, this research aims to find out the opinion of students in the Faculty of Education at the University of Granada on the sexual education training they have received and the importance of this training in their professional practice. In addition, an analysis has been carried out of the differences in each of the variables explored, according to gender.

## 2. Materials and Methods

### 2.1. Design

To analyse the existing reality on this issue, a description has been made based on the opinion of a specific population using a quantitative and exploratory research design, as we want to verify the relationship between variables at a specific moment in time.

### 2.2. Participants

The participants in this research were selected through non-probability or convenience sampling [27] in which all students were invited to participate and were taught by the researchers in this study during the 2021–2022 academic year.

The study sample consisted of 293 students from the Faculty of Education Sciences and the inter-university master’s degree in culture of peace, conflict, education and human rights at the University of Granada, Spain. Specifically, the young people were students with the master’s degree in research, social development, and socio-educational intervention (18.1%), the degree in social education (28.7%), the degree in pedagogy (23.5%), the degree in primary education (11.6%), and the degree in early childhood education (18.1%). The sample consisted of 86.3% women and 13.7% men, with a mean age of 21.51 (SD = 4.3) and with maximum and minimum values of 49 and 18 years, respectively.

### 2.3. Measuring Instruments

The instrument used for data collection was the New Brunswick Students’ Ideas about Sexual Health Education questionnaire, developed by Byers et al. [28]. The questionnaire was adapted to the context of the participants in Spain. This instrument consists of 11 questions, which analyse socio-demographic variables, the need for and importance of comprehensive sexual education, and the degree of importance of the topics to be covered in sexual education at university.

Cronbach’s Alpha statistic, with a value of α = 0.754, showed optimal reliability of the scale [29]. The questionnaire is assessed using a Likert-type scale with five response options: 1: totally disagree, 2: disagree, 3: neither agree nor disagree, 4: agree, and 5: totally agree, for the first dimension, and 1: none, 2: little, 3: indifferent, 4: quite a lot, and 5: a lot, for the second dimension.

### 2.4. Procedure

For the collection of information, the instrument was prepared to be administered at the Faculty of Education Sciences of the University of Granada (Spain). The instrument was prepared to be administered online using Google forms software. This process was carried out during the 2021–2022 academic year.

### 2.5. Data Analysis

Data analysis was carried out using the SPSS v.26 statistical package. Descriptive and inferential statistics were used with non-parametric tests, such as Mann–Whitney U test, given the results of the Kolmogórov–Smirnov normality test where a value of *p* < 0.05 was obtained, specifically 0.001. Cohen’s d was also calculated to consider the effect size.

In all cases, the statistical significance level obtained was α = 0.05.

## 3. Results

We begin by describing participants’ views on the need for and importance of comprehensive sexual education. As we can see in Table 1, 58% of the participants say that they have received poor sexual education, followed by 20.8% who acknowledge that it has been good and in contrast with 18.1% who say that they have had no education at all. Only 3.1% consider that they have received excellent sexual education.

Concerning the following dimensions of analysis, and as can be seen in Table 2, the questions “Do you think that the university should offer courses and activities that facilitate sexual education?” and “Do you think that as people and professionals in education we need comprehensive sexual education?” (M = 3.95) led to the highest agreement among the participants, followed by “Do you think that universities should address the need for sexual education?” (M = 3.92) and “Do you think that in the Faculty of Education degrees there should be a subject on sexual education?” (M = 3.88).

Regarding the dimensions with the lowest mean rating, we find “I believe that educational professionals receive proper training in sexual education” (M = 2.24), followed by “I consider sexology to be an officially recognised profession and supported by a professional association.” (M = 2.94).

With regards to the degree of importance of the topics to be dealt with in sexual education at university (Table 3), the most highly rated topic (M = 4.87) was respect, followed by education for gender equality (M = 4.75) and sexual health (M = 4.72). The lowest-rated theme is sexual pleasure (M = 4.13). All the topics are perceived by the sample as very important to receive an optimal sexual education at university.

In relation to the existence of statistically significant differences in each of the variables explored, according to sex, the Mann–Whitney U test was applied (Table 4).

In Table 4, statistically significant differences appear in the variables “Do you think universities should address the need for sexual education?” (*p* =.001), “I believe that Sexology is an officially recognised profession and supported by a professional association” (*p* = 0.018), “Would you participate in sexual education activities?” (*p* = 0.020), “Do you think there should be a subject on sexual education in the Faculty of Education?” (*p* = 0.025), and the degree of importance they assign to sexual health within sexual education at university (*p* = 0.005).

The values obtained when analysing Cohen’s d for the aforementioned variables give a value of 1.060 to the variable “I consider Sexology to be an officially recognised profession and supported by a professional association”, with a greater effect size and therefore a bigger difference between the two groups according to sex.

In all the previously mentioned items where we found significant differences between men and women, the differences are in favour of women compared to men with higher averages, except in the item “I consider sexology to be an officially recognised profession and backed by a professional association”, where the average for men is significantly higher (see Table 5).

With respect to the rest of the variables, there were no statistically significant differences.

## 4. Discussion

In health education, it is important to highlight the promotion of sexual health, defined by the Pan American Health Organisation [9] as “the experience of the continuum of physical, psychological and socio-cultural well-being related to sexuality” and which “requires an environment free of coercion, discrimination and violence”. Two strategies to promote sexual health within the framework of health education are highlighted: “providing sexual education at the school level” and “integrating sexual education into the curricula of educational institutions, as appropriate”.

Sexual education can be defined as the set of pedagogical activities using objective and complete information at the biological, psychological, and social levels to train in sexuality, developing skills as well as promoting the acquisition of knowledge, thus leading to the development of critical thinking that generates positive attitudes towards sexuality [30]. For this education to be comprehensive, it must promote human rights, advance gender equality, and improve sexual and reproductive health [5]. It is important to highlight that the lack of training in sexual education exposes young people to situations of vulnerability because they do not have the necessary tools and knowledge to act correctly, facing unhealthy sexual behaviours [31].

Sexual education should be approached as a quest for the fullness of human personal fulfilment in its relationship with other people in the world [19]. It should be based on co-educational approaches to treat people holistically [32]. Addressing sexual education in a comprehensible way in the university environment will have an impact on multiple forms of discrimination, highlighting the fight against male violence, as well as on the development of identity in a full, healthy, and conscious way [33].

Most of the participants in our study acknowledged a lack of sexual education. This lack of training for future education professionals is a problem, as they have a fundamental role in the process of training, guidance, and education, and if they do not have the appropriate knowledge and attitudes, they will not be able to play the role of sexual educators [4,34].

This study highlights the need for university education through a comprehensive sexual education subject, creating an area of knowledge related to health education, with sexual education content such as respect, education for gender equality, sexual health, sexual decision making, violence in relationships, orientation of sexual response, cultural and religious understandings of sexuality, or sexual pleasure, as a result that coincides with the research carried out by Fernández-Fernández and Calvo-González [1] or Rouco, Vargas, and Martínez [35]. Martínez et al. [36] conclude with the need to introduce sexual education in an explicit way in the educational curriculum, creating an area related to health, well-being, and quality of life, where sexual education content related to health education, education in values, environmental education, peace education, and, in general, all cross-cutting content would have a place, and universities should promote the training of education professionals as specifically as possible.

## 5. Conclusions

Understanding the importance of comprehensive sexual education for future educational professionals is fundamental to designing effective programmes for young people. In this study, the majority of respondents consider sexual education to be a recognised right, highlighting the importance of educational professionals receiving proper training in sexual education, together with the incorporation of this training at university, through courses or activities or a subject, and the inclusion of a wide range of topics (respect, education for gender equality, sexual health, sexual decision making, violence in relationships, orientation of sexual response, cultural and religious understandings of sexuality, sexual pleasure) in the curriculum.

Finally, the results of this study are contextualised in the province of Granada and, despite its limitations, it allows us to obtain information about comprehensive sexual education as a means of promoting training at university. From these limitations, we can deduce the need for further research to be able to carry out a training programme for future educational professionals.

## Figures and Tables

**Table 1:** Descriptive statistics on the degree of sexual education received.

Received Sexual Education?				
	Frequency	Percentage	Valid Percentage	Cumulated Percentage
Have had no training	53	18.1	18.1	18.1
Deficient	170	58.0	58.0	76.1
Good	61	20.8	20.8	96.9
Excellent	9	3.1	3.1	100.0
Total	293	100.0	100.0	

**Table 2:** Descriptive analyses of the need for the importance of comprehensive sexual education.

Statistics						
	N		Average	Standard Deviations	Minimum	Maximum
	Valid	Missing				
I consider that sex education is a recognized right	293	0	3.16	0.985	1	4
I consider that Sexology is an officially recognized profession and endorsed by a professional association	293	0	2.94	1.068	1	4
There are networks of institutions interested in sex education	293	0	3.35	0.826	1	4
I believe that education professionals receive organized training in sex education	293	0	2.24	0.988	1	4
Do you think universities should address the need for sex education?	293	0	3.92	0.370	1	4
Do you think that in the degrees of the Faculty of Education there should be a subject on sex education?	293	0	3.88	0.441	1	4
Do you think that the university should offer courses and activities that facilitate sexual education?	293	0	3.95	0.300	1	4
Do you think that as people and education professionals we need comprehensive sexual education?	293	0	3.95	0.281	1	4
Would you participate in sex education activities?	293	0	3.86	0.480	1	4

**Table 3:** Degree of the importance of the topics to be dealt with in sexual education at university.

Statistics				
	N		Average	Standard Deviations
	Valid	Missing		
Sexual pleasure	293	0	4.13	0.961
Gender equality education	293	0	4.75	0.586
Sexual response orientation	293	0	4.53	0.770
Respect	293	0	4.87	0.431
Sexual decision making	293	0	4.67	0.742
Sexual Health	293	0	4.72	0.700
Violent relationships	293	0	4.64	0.979
Cultural and religious understandings of sexuality	293	0	4.32	0.890

**Table 4:** Significant differences by Mann–Whitney U test.

Test Statistics ^a^				
	U de Mann–Whitney	W de Wilcoxon	Z	Sig. Asin. (Bilateral)
Have I received sex education?	4732.500	36,863.500	−0.740	0.459
I believe that sex education is a recognized right	4687.000	36,818.000	−0.816	0.415
I consider that Sexology is an officially recognized profession and endorsed by a professional association	3941.000	36,072.000	−2.372	0.018
There are networks of institutions interested in sex education	4906.000	37,037.000	−0.345	0.730
I believe that education professionals receive organized training in sex education	4419.500	36,550.500	−1.349	0.177
Do you think universities should address the need for sex education?	4063.000	4883.000	−5.086	<0.001
Do you think that in the degrees of the Faculty of Education there should be a subject on sex education?	4519.000	5339.000	−2.244	0.025
Do you think that the university should offer courses and activities that facilitate sexual education?	4822.000	5642.000	−1.520	0.129
Do you think that as people and education professionals we need comprehensive sexual education?	4738.000	5558.000	−1.813	0.070
Would you participate in sex education activities?	4458.500	5278.500	−2.333	0.020
Sexual pleasure	4513.500	5333.500	−1.177	0.239
Education for gender equality	4419.000	5239.000	−1.897	0.058
Orientation of sexual response	4455.000	5275.000	−1.445	0.148
Respect	5022.000	5842.000	−0.150	0.881
Sexual decision making	4435.000	5255.000	−1.732	0.083
Sexual health	4121.000	4941.000	−2.815	0.005
Relationship violence	4588.000	5408.000	−1.460	0.144
Cultural and religious understandings of sexuality	4324.000	5144.000	−1.639	0.101

^a^ grouping variable: sex.

**Table 5:** Average range according to sex.

Rangos				
	Sex	N	Mean Range	Sum of Ranks
Have I received sex education?	Woman	253	145.71	36,863.50
	Men	40	155.19	6207.50
	Total	293		
I believe that sex education is a recognized right	Woman	253	145.53	36,818.00
	Men	40	156.32	6253.00
	Total	293		
I consider that Sexology is an officially recognized profession and endorsed by a professional association	Woman	253	142.58	36,072.00
	Men	40	174.98	6999.00
	Total	293		
There are networks of institutions interested in sex education	Woman	253	146.39	37,037.00
	Men	40	150.85	6034.00
	Total	293		
I believe that education professionals receive organized training in sex education	Woman	253	144.47	36,550.50
	Men	40	163.01	6520.50
	Total	293		
Do you think universities should address the need for sex education?	Woman	253	150.94	38,188.00
	Men	40	122.08	4883.00
	Total	293		
Do you think that in the degrees of the Faculty of Education there should be a subject on sex education?	Woman	253	149.14	37,732.00
	Men	40	133.48	5339.00
	Total	293		
Do you think that the university should offer courses and activities that facilitate sexual education?	Woman	253	147.94	37,429.00
	Men	40	141.05	5642.00
	Total	293		
Do you think that as people and education professionals we need comprehensive sexual education?	Woman	253	148.27	37,513.00
	Men	40	138.95	5558.00
	Total	293		
Would you participate in sex education activities?	Woman	253	149.38	37,792.50
	Men	40	131.96	5278.50
	Total	293		
Sexual pleasure	Woman	253	149.16	37,737.50
	Men	40	133.34	5333.50
	Total	293		
Education for gender equality	Woman	253	149.53	37,832.00
	Men	40	130.98	5239.00
	Total	293		
Orientation of sexual response	Woman	253	149.39	37,796.00
	Men	40	131.88	5275.00
	Total	293		
Respect	Woman	253	147.15	37,229.00
	Men	40	146.05	5842.00
	Total	293		
Sexual decision making	Woman	253	149.47	37,816.00
	Men	40	131.38	5255.00
	Total	293		
Sexual health	Woman	253	150.71	38,130.00
	Men	40	123.53	4941.00
	Total	293		
Relationship violence	Woman	253	148.87	37,663.00
	Men	40	135.20	5408.00
	Total	293		
Cultural and religious understandings of sexuality	Woman	253	149.91	37,927.00
	Men	40	128.60	5144.00
	Total	293		

## Data Availability

The data presented in this study are available upon request from the corresponding author.
